# Understanding flash flooding in the Himalayan Region: a case study

**DOI:** 10.1038/s41598-024-53535-w

**Published:** 2024-03-25

**Authors:** Katukotta Nagamani, Anoop Kumar Mishra, Mohammad Suhail Meer, Jayanta Das

**Affiliations:** 1 Centre for Remote Sensing and Geoinformatics, Sathyabama Institute of Science and Technology, Chennai, India; 2 Office of Director General of Meteorology, India Meteorological Department, Ministry of Earth Science, SATMET Division, Mausam Bhavan, New Delhi, 110003 India; 3 Department of Geography, Rampurhat College, PO- Rampurhat, Birbhum, 731224 India

**Keywords:** Natural hazards, Climate sciences, Cryospheric science

## Abstract

The Himalayan region, characterized by its substantial topographical scale and elevation, exhibits vulnerability to flash floods and landslides induced by natural and anthropogenic influences. The study focuses on the Himalayan region, emphasizing the pivotal role of geographical and atmospheric parameters in flash flood occurrences. Specifically, the investigation delves into the intricate interactions between atmospheric and surface parameters to elucidate their collective contribution to flash flooding within the Nainital region of Uttarakhand in the Himalayan terrain. Pre-flood parameters, including total aerosol optical depth, cloud cover thickness, and total precipitable water vapor, were systematically analyzed, revealing a noteworthy correlation with flash flooding event transpiring on October 17th, 18th, and 19th, 2021. Which resulted in a huge loss of life and property in the study area. Contrasting the October 2021 heavy rainfall with the time series data (2000–2021), the historical pattern indicates flash flooding predominantly during June to September. The rare occurrence of October flash flooding suggests a potential shift in the area's precipitation pattern, possibly influenced by climate change. Robust statistical analyses, specifically employing non-parametric tests including the Autocorrelation function (ACF), Mann–Kendall (MK) test, Modified Mann–Kendall, and Sen's slope (q) estimator, were applied to discern extreme precipitation characteristics from 2000 to 201. The findings revealed a general non-significant increasing trend, except for July, which exhibited a non-significant decreasing trend. Moreover, the results elucidate the application of Meteosat-8 data and remote sensing applications to analyze flash flood dynamics. Furthermore, the research extensively explores the substantial roles played by pre and post-atmospheric parameters with geographic parameters in heavy rainfall events that resulted flash flooding, presenting a comprehensive discussion. The findings describe the role of real time remote sensing and satellite and underscore the need for comprehensive approaches to tackle flash flooding, including mitigation. The study also highlights the significance of monitoring weather patterns and rainfall trends to improve disaster preparedness and minimize the impact of flash floods in the Himalayan region.

## Introduction

The most significant challenges affecting a country's long-term social, economic, and environmental well-being stem from natural disasters. This includes extreme hydro-meteorological events like cloudbursts and excessive rainfall, which, due to their severe complications and intensity, have become a focal point of research, particularly in mountainous areas. The exploration of these events is crucial for developing strategies to mitigate their impact for mountainous region^1^. In the Himalayan context, the discernment of topographical intricacies assumes paramount importance due to their potential rapid escalation into calamitous events^2^. Consequently, a comprehensive understanding of hydrological challenges and water resource resilience becomes imperative, as these phenomena manifest in diverse catastrophic forms^3^. To delineate and analyze these hydrological challenges and resilience, hydrological modeling emerges as a crucial tool. The efficacy of such modeling is contingent upon the utilization of high-resolution geospatial data, particularly within the Soil and Water Assessment Tool (SWAT) framework. This integration enhances precision in water resource management, addressing the intricacies posed by the challenging Himalayan terrain^4^. This aligns with the study of Patel et al. 2022^5^, who concentrate on the 2013 Uttarakhand flash floods, underlining the importance of hydrological assessments and the development of disaster preparedness strategies in the region. The catastrophic nature of flash floods caused by cloud bursts and landslides in mountainous regions is highlighted as the most devastating natural disaster^6^. Instances of such disasters precipitate multifaceted consequences, encompassing loss of life, infrastructural degradation, and disruption of financial operations. Mitigating these adversities necessitates the systematic monitoring and analysis of flood events. A historical examination underscores the pivotal role of floods, emerging as the foremost impactful natural calamity, with an annual average impact on over 80 million individuals globally over the past few decades. The substantial global impact, as evidenced by floods contributing to annual economic losses exceeding US$11 million worldwide^7^, is further underscored by the difficulty in collecting information on land use, topography, and hydro-meteorological conditions. Anticipating an increased frequency of precipitation extremes and associated flooding in Asia, Africa, and Southeast Asia in the coming decades, this challenge has prompted a debate on the necessary adaptations in flood management policies to address this evolving reality^8^. India, facing the highest flood-related fatalities among Asian countries^9,10^, encounters heightened vulnerability to disaster threats. This susceptibility is further exacerbated by the country's extensive geographic variability, making the development and implementation of a climate response strategy considerably more challenging^11^.

The Indian Himalayan Region, being crucial to the national water, energy, and food linkage due to its variety of political, economic, social, and environmental systems, is uniquely vulnerable to hydro-meteorological catastrophes, including floods, cloudbursts, glacier lake eruptions, and landslides^12–15^. During monsoon season the cloud burst is increasing in the Himalayan region.This phenomenon is closely tied to the unique climatic conditions prevalent in the Himalayas during this period. Monsoons in this region bring intense and sustained rainfall, characterized by the convergence of moisture-laden air masses, especially from the Bay of Bengal, attributing to landslides, debris flows, and flash flooding^16^. These result in significant loss of life, property, infrastructure, agriculture, forest cover, and communication systems^17^. In 2013, the Himalayan state of Uttarakhand experienced devastating floods and landslides due to multiple heavy rainfall spells^17,18^. On February 7th, 2021, a portion of the Nanda Devi glacier in Uttarakhand's Chamoli district broke off, causing an unanticipated flood^19–21^. During this sudden flood, 15 people were killed, and 150 went missing. These disasters have disrupted the Himalayan ecology in several states, including Uttarakhand, and the cause and magnitude of these disasters have been made worse by human activities, including building highways, dams, and deforestation^22^. When we check the flood record of Uttarakhand, Himalaya, the area has experienced catastrophes during 1970, 1986, 1991, 1998, 2001, 2002, 2004, 2005, 2008, 2009, 2010, 2012, 2013, 2016, 2017, 2019, 2020, and 2021, making them among the most significant natural disasters to have struck Uttarakhand^16,21^.

The rising trend of the synoptic scale of Western Disturbance (WD) activity and precipitation extremes over the Western Himalayan (WH) region during the last few decades is the result of human-induced climate change, and these changes cannot be fully explained by natural forcing alone. This phenomenon is observed over the large expanse of the high-elevation eastern Tibetan Plateau, where a higher surface warming in response to climate change is noted compared to the western side^22,23^. Since the Industrial Revolution, the Himalaya and the Tibetan plateau have warmed at an increased rate of 0.2 degrees each decade (1951–2014)^24^. In the Himalayan region, the mean surface temperature has increased by almost 0.5˚C during 2000–2014. This alteration in climate (temperature) has resulted in a decrease in the amount of apples produced in low-altitude portions of the Himalaya. The warming of the planet is directly responsible for these effects. The Himalayan region has experienced a decline in pre-monsoon precipitation towards the end of the century, leading to new societal challenges for local farmers due to the socioeconomic shifts that have taken place^25^. Simultaneously, there has been an increase in the highest recorded temperature observed throughout the monsoon season. In tandem with heightened levels of precipitation, an elevation in the maximum attainable temperature has the potential to amplify the occurrence of torrential rainfall events during the monsoon season^26^. This long-term change in atmospheric parameters, known as climate change, may affect river hydrology and biodiversity. The associated shifts in climate pose a significant risk to hydropower plants if certain climate change scenarios materialize. As part of this broader context, the dilemma of spring disappearance should be thoroughly analyzed to provide scientific, long-term remedies and mitigation strategies for potential hydrogeological disasters. This is crucial due to the observed increase in the frequency of landslides, avalanches, and flash floods in recent years^24^.

El Niño–Southern Oscillation (ENSO) and Equatorial Indian Ocean Oscillation (EQUINOO) play a crucial role in the teleconnection of India's Monsoon, as well as in determining rainfall patterns and the occurrence of flash floods across different regions of India. At a regional level, a study was conducted to examine the impact of various types of climatic fluctuations on the onset dates of the monsoon. Northern India, specifically northern northwest India, referred to as SR15, consistently experiences a delayed start to its seasons, regardless of the climatic phase^27^. The occurrence of significant anomalies in sea surface temperatures (SST) in the tropical Pacific region, associated with ENSO and EQUINOO, is accompanied by large-scale tropical Sea Level Pressure (SLP) anomalies related to the Southern Oscillation (SO)^28,29^. The Equatorial Indian Ocean Oscillation (EIO) represents the oscillation between these two states, manifested in pressure gradients and wind patterns along the equator (EQUINOO).

 The negative anomaly of the zonal component of surface wind in the equatorial Indian Ocean region (60°–90°, 2.5° S—2.5° N) is the foundation for the EQUINOO index^30^. Additionally, they demonstrated that between 1979 and 2002, any season with excessive rainfall or drought could be "explained" in terms of the favorable or unfavorable phase of either the EQUINOO, the ENSO, or both. For instance, in 1994, EQUINOO was favorable, but ENSO was negative, resulting in above-average rainfall in India. Conversely, ENSO was favorable, EQUINOO was unfavorable between 1979 and 1985, and India saw below-average rainfall. They, therefore, proposed that by combining those two climate indices, it would be possible to increase the predictability of rainfall during the Indian monsoon. The quantity of rainfall throughout a storm event that might cause a significant discharge in a particular river segment is known as a "rainfall threshold"^31,32^. Different techniques, indicators, and predictor variables can be used to derive rainfall thresholds. There are four categories of methodology: empirical, hydrological/hydrodynamic, probabilistic, and compound approaches. Empirical rainfall thresholds are among the most popular methods for constructing EWS in local, regional, and national areas^33–35^. Empirical methods use historical flood reports and rainfall amounts to perform a correlation analysis linking the frequency of event to the amount and length of essential precipitation^36–38^. Several empirical rainfall threshold curves may be found in literature from various countries^32,39–41^. Although this research concentrated on various shallow landslides and mudflows, flash flood risk systems can be set using actual rainfall thresholds^42^. Similarly, the principles of the Flood Risk Guideline (FFG) method serve as the foundation for hydrogeological precipitation limits^30,41,43,44^. The fundamental concept of FFG is to use reverse hydrologic modelling to identify the precipitation that produces the slightest flood flow at the basin outlet. Alerts are sent out whenever the threshold is exceeded for a specific time for the real-time actual daily rainfall or the precipitation forecast. This method needs data on precipitation collected using radar or real-time rainfall sensors^45,46^. Other threshold approaches for rainfall, however, require the same data. The modelling of various synthetic photographs, regionally dispersed models, and the prior soil moisture status have all been incorporated into the FFG, which is widely used worldwide^46^. Hydraulic models have been developed recently, allowing the threshold to be determined by the canal design, features, and the link between the achieved water table and the inundated area^47,48^.

Recent flood events underscore the inadequacy of relying solely on structural safeguards for comprehensive protection against such catastrophes. The imperative for an effective flood management approach becomes paramount to preemptively mitigate these calamities and ensure sustainable safety measures. The present study generates rainfall product that uses real-time satellite data from Meteosat-8 to summarize the significant short-lived localised multiple rainfall events that result flash flooding in the Nainital, Uttrakhand, during October 2021^48^. This method was utilized to investigate the flood events over J&K 2014^49^. Rajasthan in 2019^50^ and Bihar and Assam in 2019^51^. This study introduces a pioneering approach by precisely measuring the peak rainfall hours and correlating them with daily rainfall, elucidating their direct correlation with flash flooding in the study area. A distinctive feature of this research is its integration of time series rainfall data with socioeconomic metrics to underscore the significant damage caused by a major flash flood incident. The exploration of the role of sheer slope in flooding provides a unique angle to flood dynamics. Additionally, the study delves into pre-atmospheric parameters specific to the study area that played a pivotal role in initiating flash flooding. By shedding light on these intricate details, this study establishes itself as a trailblazer in disaster mitigation strategies, emphasizing its pivotal role in advancing our understanding of flash flood dynamics and fortifying disaster response frameworks.

## Study area

**Figure 1:**
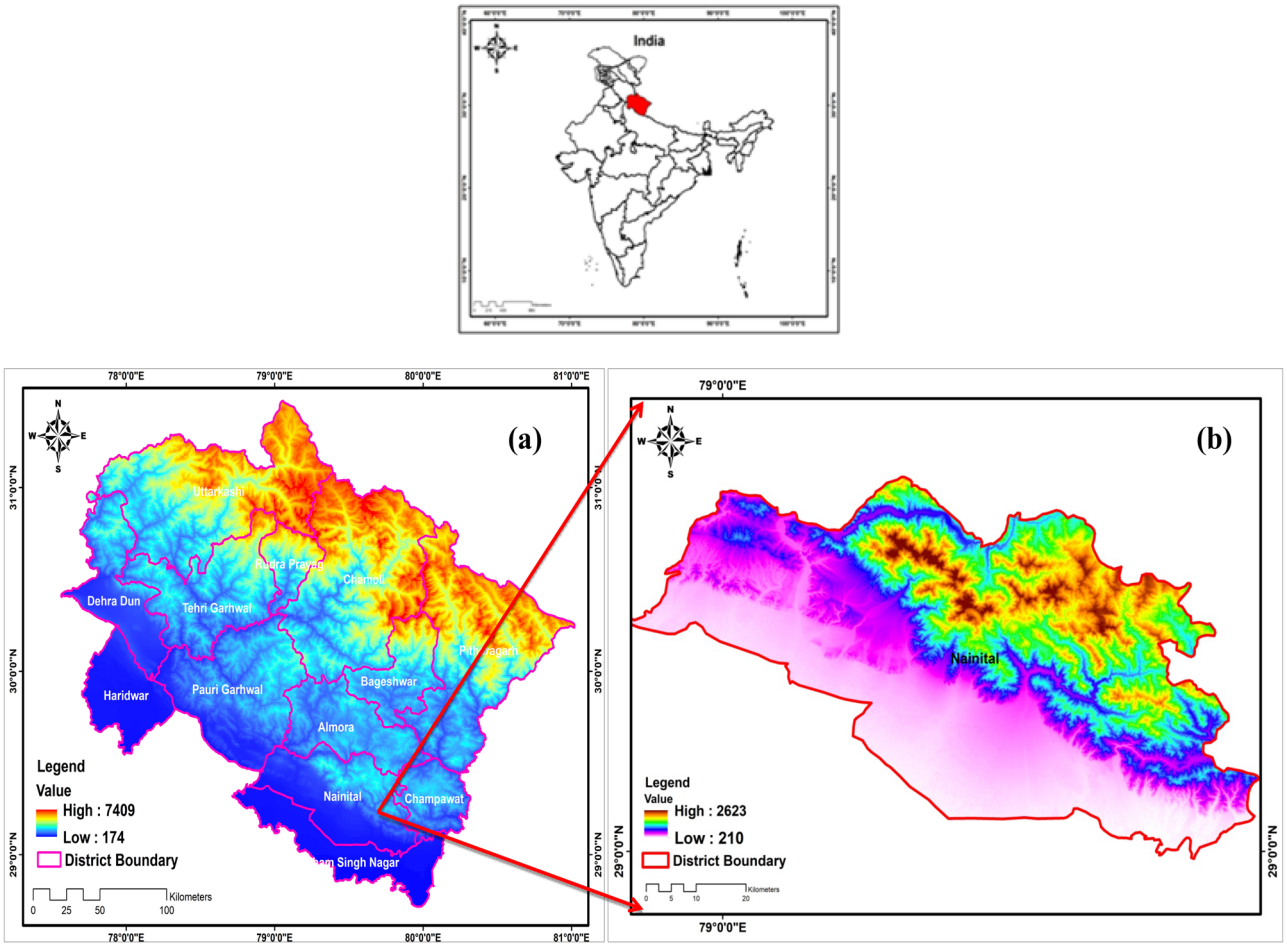
(**a**) Showing Uttarakhand North western Himalayan state of India (**b**) Nainital district of Uttarakhand with Digital elevation model.

The economic and climatic conditions of India are intricately linked to the region of Himalaya, renowned for its delicate ecosystems and geological intricacies^52^. Spanning a vast area, the Indian, Himalaya is among the recent mountain ranges on the surface of earth, marked by the study delves into the vulnerability of the region of Himalaya, examining the intricate interplay of geographical and atmospheric parameters in flash flood occurrences. The area has susceptibility to geological hazards, topographical nuances, biodiversity, and water resource dynamics^53^. Geographically positioned between latitudes 28.44° to 31.28°N and longitudes 77.35° to 81.01°E, with elevations ranging from 7409 to 174 m, Uttarakhand, depicted in Fig. 1, covers 53,483 square kilometers. Approximately 64% of the land is forested, and 93% is mountainous terrain, bordered by Himachal Pradesh, Uttar Pradesh, China, and Nepal. Serving as the source of major rivers, the state encompasses six significant basins: Yamuna, Alaknanda, Ganga, Kali, Bhagirathi, and Ramganga. Data analysis utilized Shuttle Radar Topographic Mission information obtained from Earth Explorer (https://earthexplorer.usgs.gov) via Arc GIS Version 10.5, as shown in Fig. 1.

## Climate characteristics

The climate of study area exhibits notable variations, ranging from humid subtropical conditions in the Terai region to tundra-like environments in the Greater Himalaya. Substantial transformations occur across the landscape, with high altitudes housing glaciers and lower elevations supporting subtropical forests. Annual precipitation contributes nourishing snowfall to the Himalaya, particularly above 3000 meters^54^. Temperature variations are influenced by elevation, geographical position, slope, and topographical factors. In March and April, southern areas experience average maximum temperature between 34 °C and 38 °C, with average minimum temperatures ranging from 20 °C to 24 °C. Temperatures peak in May and June, reaching up to 42 °C in the lowlands and around 30 °C at elevations exceeding two kilometers. A decline in temperatures begins in late September, reaching their lowest points in January and early February, with January being the coldest month. Southern regions and river valleys witness an average maximum temperature of approximately 20 °C and an average minimum temperature of about 6 °C, while elevation of 2 km above sea level range from 10 °C to 12 °C^55^.

## Materials and methodology

Radar is used to collect the rainfall observation remotely. A rain gauge is a conventional method located on the ground for recording rainfall depth in millimeters. Radar systems and rain gauges are standard equipment for tracking significant rainfall events. If there is a widespread, uniform network of rain gauges, it is possible to monitor rainfall accurately unfortunately, there is no such system in Nainital, Uttarakhand, or other parts of India. With the diverse topography of Nainital, Uttrakhand, it is challenging to observe accuracy for extreme rainfall events using radar and rain gauge stations. Satellite observation is the only tool available for monitoring these events. The extreme rainfall event over Nainital, Uttarakhand, was tracked in this study using hourly measurements of rainfall from Meteosat-8 geostationary satellite data. Hourly rainfall measurement was estimated at five kilometres by integrating observation from the Meteosat-8 satellite with space-borne precipitation Radar (PR) from the tropical rainfall measuring mission (TRMM). To estimate rainfall using Meteosat-8 IR and water vapour (WV) channels at 5 km resolution, we have employed the rain index-based technique created by Mishra, 2012^48^. The techniques use TRMM (Tropical rainfall Measuring Mission), space-borne precipitation radar (PR), and Meteosat-8 multispectral satellite data to create the rain analysis. The technique uses Infrared and water vapour observation from Meteosat-8 on 17, 18 and 19 October 2021 to estimate the amount of rainfall over the Nainital, Uttarakhand. By using the infrared (IR) and water vapour (WV) channel observations from Meteosat-8, a new rain index (RI) was computed. The procedure for calculating the rain index is as follows. Non-rainy clouds are filtered out using spatial and temporal gradient approach and brightness temperature from thermal Infrared (TIR) and WV are collocated against rainfall from precipitation radar (PR) to derive non-rainy thresholds of brightness temperature from TIR and WV channels. Now TIR and WV rain coefficient is computed by dividing the brightness temperature from TIR and WV channels with non-rainy thresholds. The TIR ad WV, rain coefficient product, is defined as the rain index (RI). RI is collocated against rainfall from PR to develop a relationship between rainfall and RI using large data sets of heavy rainfall events during the monsoon season of multiple years. The following equation is developed between rain rate (RR) and RI:

1 $${\text{RR }} = a + \, \left( {b \times {\text{ RI}}^{c} } \right).$$

Finally, the rainfall rate (RR) is calculated using Eq. (1). For the Indian subcontinent, a, b, and c are calculated as a = 8.4969, b = 2.7362, and c = 4.27. Using RI generated from Meteosat-8 measurements, this model may be used to estimate hourly rainfall.

The current equation (I) was verified using observations from a strong network of ground-based rain gauges. Hourly rain gauge readings over India during the south-west monsoon season were observed to have a correlation coefficient of 0.70, a bias of 1.37 mm/h, a root mean square error of 3.98 mm/h, a chance of detection of 0.87, a false alarm ratio of 0.13, and a skill score of 0.22^48^. The method used by Mishra^48^ outperformed other methods for examining the diurnal aspects of heavy rain over India compared to currently available worldwide rainfall statistics. If both satellite spectral responses to the channels used to produce the rain signatures are similar, the equation developed to estimate rainfall using the rain signature from one satellite can also be used to estimate rainfall using the rain signature from another satellite.

Within the framework of this investigation, Meteosat-8 Second Generation (MSG) measurements were harnessed to scrutinize rainfall characteristics with a heightened focus on fine geographical and temporal scales. Employing the mentioned technique facilitated the calculation of spatial rainfall distribution, as well as the meticulous quantification of hourly and daily rainfall. Subsequently, a comprehensive analysis of cumulative rainfall was conducted, unraveling nuanced patterns and trends within the meteorological data. Following an in-depth examination of intense rainfall episodes, the atmospheric datasets, incorporating cloud optical thickness, total precipitable water vapor, and aerosol optical depth, were procured from Modern-Era Retrospective Analysis for Research and Applications, the National Centers for Environmental Prediction (NCEP), and the National Centre for Atmospheric Research (NCAR). These datasets underwent meticulous scrutiny to unravel the intricate interconnections between atmospheric parameters and heavy rainfall, specifically flash flooding, across the study area. The central objective was to decipher the meteorological conditions catalyzing the genesis of a low-pressure system, subsequently triggering heightened convective activities. To comprehend the dynamics of aerosols within the study domain, trajectory analysis through HYSPLIT was implemented, elucidating trajectories and dispersion patterns of aerosols for comprehensive insights. To comprehensively comprehend episodes of heavy rainfall in the Nainital region of Uttarakhand, particularly during the flash flooding events of October 2021, this study systematically delves into pre-flood parameters. The investigation focuses on Nainital and systematically analyzes time series rainfall data (Modern-Era Retrospective Analysis for Research and Applications) spanning from 2000 to 2021. Monthly rainfall for each year and the long-term mean (accumulated rainfall) were meticulously calculated. Robust statistical tests applied to the time series data unveiled trends, indicating a non-significant increase overall, except for a notable decrease in July. The study further integrates Shuttle Radar Topography Mission (SRTM) topographic data and the total number of cloud burst events (https://dehradun.nic.in/) to elucidate the role of elevation in cloud burst occurrences. Exploring the relationship between elevation, annual rainfall, and maximum temperature, the research establishes critical links between heavy rainfall episodes, flash flooding, and associated loss of lives from 2010 to 2022. The study strategically correlates these aspects with time-series data, presenting instances of heavy rainfall and rapid-onset flooding. Utilizing Meteosat-8 data and remote sensing, our research pioneers dynamic flash flood analysis, shedding light on the pivotal roles played by atmospheric and geographic parameters. The time series precipitation data, spanning from 2001 to 2021, underwent rigorous trend analysis employing statistical methodologies, including Autocorrelation function (ACF), Mann–Kendall (MK) test, Modified Mann–Kendall test, and Sen's slope (q) estimator. These analyses were conducted to elucidate and characterize the prevailing trends within the rainfall dataset over the specified temporal interval.

## Autocorrelation function (ACF)

Autocorrelation or serial dependency is one of the severe drawbacks for analyzing and detecting trends of time series data. The existence of autocorrelation in the time series data may affect MK test statistic variance (S)^56,57^. Hence, the ACF at lag-1 was calculated using the following equation.

2 $$r_{k} = \frac{{\sum _{{k = 1}}^{{N - K}} \left( {x_{p} - \overline{{x_{p} }} } \right)\left( {x_{{p + k}} - \overline{{x_{{p + k}} }} } \right)}}{{\left[ {\sum\limits_{{k = 1}}^{{n - k}} {\left( {x_{p} - \overline{{x_{p} }} } \right)^{2} \left( {x_{{t + k}} - \overline{{x_{{p + k}} }} } \right)^{2} } } \right]^{{0.5}} }},$$

where, $${r}_{k}$$ denotes the ACF (autocorrelation function) at lag k, $${x}_{t}$$ and $${x}_{p}$$ is the utilized rainfall data, $$\overline{x}$$ denotes the mean of utilized data $$\left({x}_{p}\right)$$,$$N$$ signify the total length of the time-series ($${x}_{p})$$, k refers to the maximum lag.

## Mann–Kendall (MK) test

In hydroclimatic investigations, the MK test is extensively employed for evaluating trends^58–60^. The-MK test^61,62^ was conferred by the World-Meteorological-Organization (WMO), which has a number of benefits^63^. The following equations can be used to construct MK test-statistic

3 $$S=\sum_{i=1}^{n-1} \cdot \sum_{j=i+1}^{n} {\text{sign}}\left({x}_{p}-{x}_{q}\right)$$

In Eq. (5), n denotes the size of the sample, whereas ${x}_{p}$ and-${x}_{q}$ denote consecutive data within a series.

4 $${\text{sign}}\left({x}_{p}-{x}_{q}\right)=\left\{\begin{array}{ll}+1& \text{ when }\left({x}_{p}-{x}_{q}\right)>0\\ 0& \text{ when }\left({x}_{p}-{x}_{q}\right)=0\\ -1& \text{ when }\left({x}_{p}-{x}_{q}\right)<0\end{array}\right.$$

The variance of $S$ is assessed in the following way

5 $${\text{Var}}\left(S\right)=s\left(n-1\right)\left(2n+5\right)-\sum_{p-1}^{q}{t}_{p}\left({t}_{p}-1\right)\left(2{t}_{p}+5\right)/18$$

whereas ${t}_{p}$ and $q$ denotes the number of ties for the ${p}^{th}$ value. Equation (9) shows how to calculate Z statistic, the standardized-test for the MK test-(Z)

6 $$Z=\left\{\begin{array}{ll}\frac{s-1}{\sqrt{{\text{Var}}(S)}}& \text{ when }S>0\\ 0& \text{ when }S=0\\ \frac{s+1}{\sqrt{{\text{Var}}(S)}}& \text{ when }S<0\end{array}\right.$$

The trend's direction is indicated by the letter Z. A negative Z value specifies a diminishing trend and vice versa. The null hypothesis of no trend will be rejected when the absolute value of Z would be greater than 2.576 and 1.960 at 1% and 5% significant level.

## Modified Mann–Kendall test

Hamed and Rao (1998)^64^ introduced the modified MK test for auto-correlated data. In the case of auto-correlated data, variance (s) is underestimated^65^; hence, the following correction factor $\left(\frac{n}{{n}_{e}^{*}}\right)$ is proposed to deal with serially dependency data.

7 $$VAR\left(S\right)=\left(\frac{n\left(n-1\right)\left(2n+5\right)}{18}\right).\left(\frac{n}{{n}_{e}^{*}}\right)$$

8 $$\left(\frac{n}{{n}_{e}^{*}}\right)=1+\left(\frac{2}{{n}^{3}-3{n}^{2}+2n}\right)\sum_{f=1}^{n-1}\left(n-f\right)\left(n-f-1\right)\left(n-f-2\right){ \rho }_{e}\left(f\right)$$

where $n$ is the total number of observations and ${\rho }_{e}\left(f\right)$ denotes the autocorrelation function of the time series, and it is estimated using the following equation

9 $$\rho \left(f\right)=2{\text{sin}}\left(\frac{\pi }{6}{ \rho }_{e}\left(f\right)\right).$$

## Sen's slope (q) estimator

Sen^66^ proposed the non-parametric technique to obtain the quantity of trends in the data series. The Sen’s slope estimator can calculate in the time series from N pairs of data using this formula

10 $${Q}_{i}=\frac{{x}_{n}-{x}_{m}}{n-m}, i=\mathrm{1,2}, 3\dots \dots ..N, n>m$$

where ${Q}_{i}$ refers to the Sen’s slope estimator,${x}_{n}$ and ${x}_{m}$ are scores of times $n$ and $m$, respectively.

## Results and discussion

**Figure 2:**
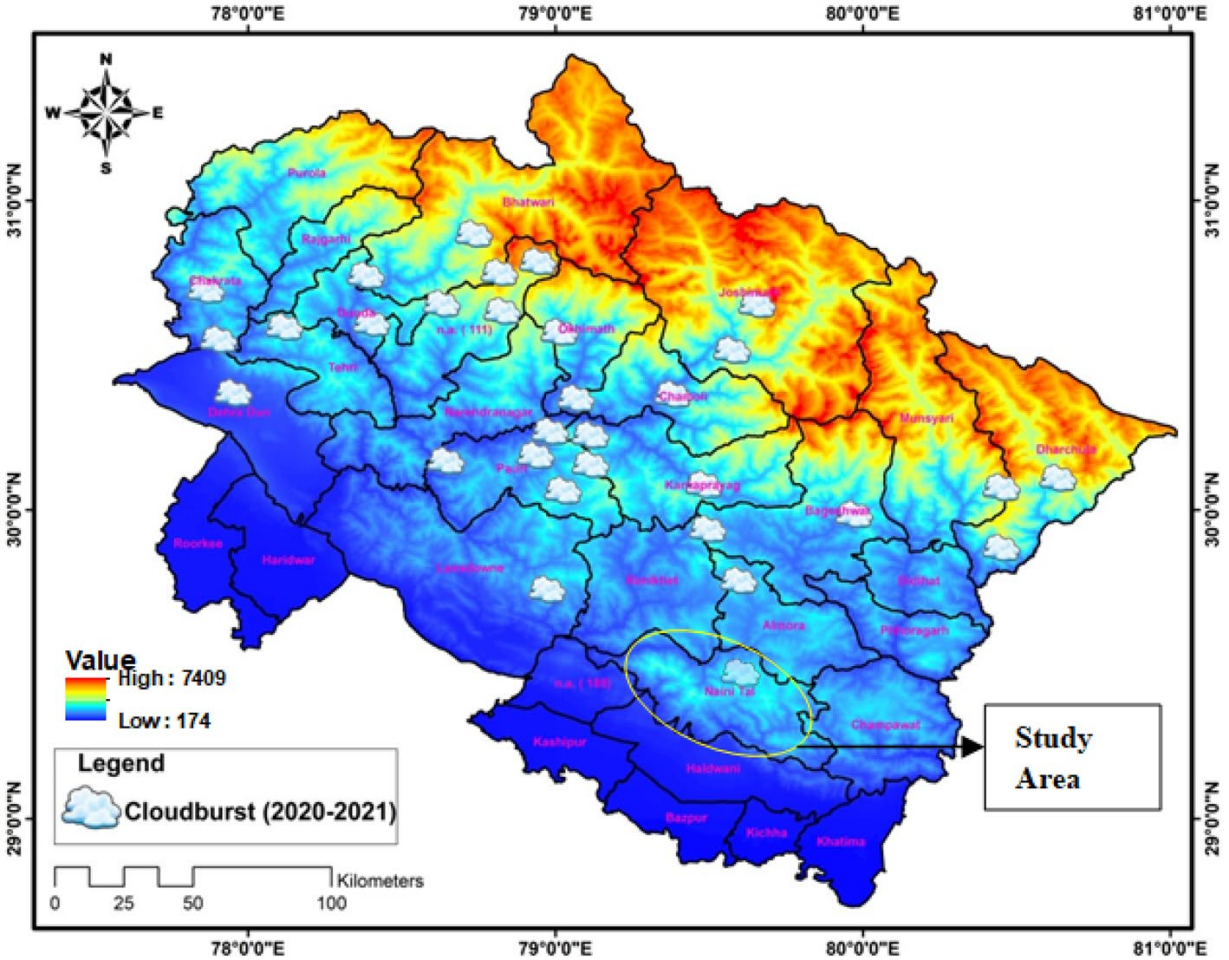
Location map of cloudbursts hit area from 2020 to 2021 over Uttrakhand.

The Himalaya, renowned for their massive size and elevated altitude, possess distinctive geological characteristics that render them vulnerable to sudden and intense floods^67^. These rapid floods are the outcome of a combination of natural and human factors, including geological movements, glacial lakes, steep topography, deforestation, alterations in land usage, and the monsoon season^68^. In the Himalayan region, the primary trigger for these abrupt floods is often linked to instances of cloud bursts accompanied by heavy rainfall episodes^69^. This study aims to provide insight into historical and recent instances of significant rainfall that have resulted in flash floods, while also examining the relationship between these events with atmospheric and other relevant factors. The study also elaborates on the discussion on flash flooding on the 17th, 18 and 19 October 2021. In Fig. 2 we have illustrated the elevation and cloud burst events that occurred between 2020 and 2021 across different districts in Uttarakhand, Himalaya. The elevation map (Fig. 2), was generated by Arc GIS 10.5. Using cloud burst data from (https://dehradun.nic.in/). After statistical analyses, the same data was imported to Arc GIS 10.5 and was shown in the form of Fig. 2**.** The figure underscores that the northern areas, located within the central portion of Uttarakhand, witnessed a higher frequency of cloud bursts compared to the southern areas. The observed divergence, attributed to steeper slopes in the northern region as opposed to the southern region, is further complemented by an intriguing revelation in our study^70^. Specifically, we noted significantly fewer cloud burst events in the areas of both lower and sharply higher elevations during the period of 2020–2021, particularly when compared with the occurrences at medium elevations from (1000 to 2500)m illustrated in Fig. 2. Thus, emphasizing a noteworthy and substantiated relationship between cloud bursts and elevation^70^.

Within the specified timeframe, a total of 30 significant cloudburst incidents were documented during 2020–2021, with 17 of these incidents transpiring in 2021. Among the districts, Uttarkashi recorded the highest number of cloudburst occurrences (07), trailed by Chamoli with 05 incidents, while Dehradun and Pithoragarh each registered 04 instances. Rudraprayag accounted for 03 incidents, whereas Tehri, Almora, and Bageshwar each reported 01 cloudburst occurrence, according to reports from the Dehradun District Administration and the India Meteorological Department in 2021.

**Figure 3:**
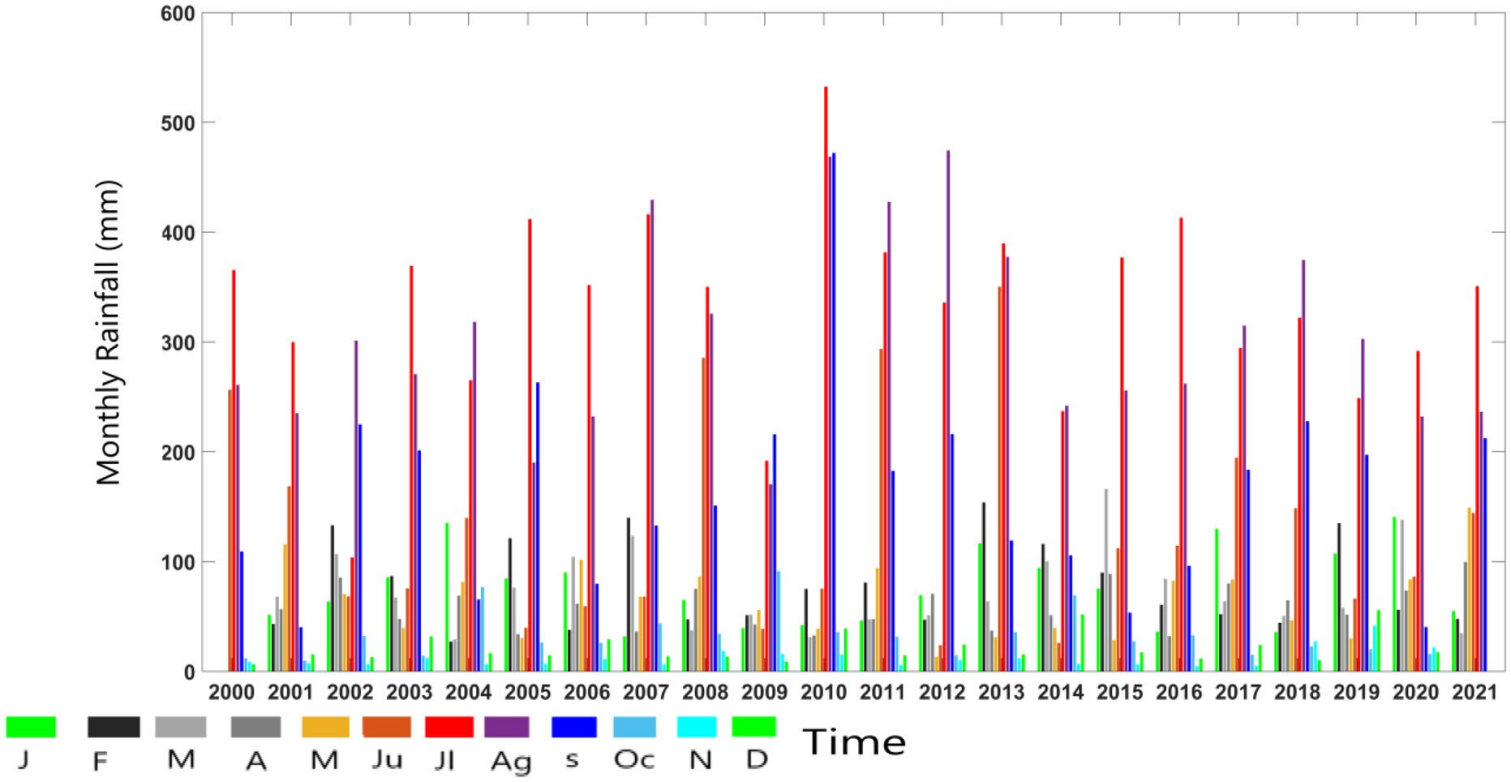
Time series monthly rainfall of study area. J(January),F(February),M(March),A(April),M(May),Ju(June)Jl(July),Ag(August), S(September),Oc(October), N(November), D(December).

Due to high topography, the area has faced many flash flood events in history. Figure 3 presents a graphical representation of the total monthly rainfall data for the Nainital district in Uttarakhand from 2000 to 2021. The graph reveals the amount of rainfall received each month throughout this period. A noteworthy observation from the graph is that most of the years between 2000 and 2021 experienced substantial rainfall, with the majority surpassing 300 mm. However, 2010 is an exceptional case of rainfall in the Nainital area. The region received an astounding 500 mm monthly rainfall during this particular year. This extraordinary amount of rainfall was unprecedented and broke the records of the last few decades. Such a significant monthly rainfall level had not been observed in the region for quite some time. The spike in rainfall during 2010 might have considerably impacted the local environment, water bodies, and overall hydrological conditions in the area. Given the intensity of the rainfall, It could have caused flooding, landslides, and other related hazards. The data presented in Fig. 3 is crucial for understanding the long-term trends and patterns of rainfall in Nainital over the past two decades. In Fig. 3, another intriguing aspect emerges, shedding light on the fact that the South-west monsoon exhibits its peak rainfall during the months of June, July, August, and September across the study area.(https://mausam.imd.gov.in/Forecast/mcmarq/mcmarq_data/SW_MONS OON_2022_UK.pdf).The region could be subject to recurring heavy rainfall episodes, potentially resulting in flash flooding over specific temporal intervals.

**Figure 4:**
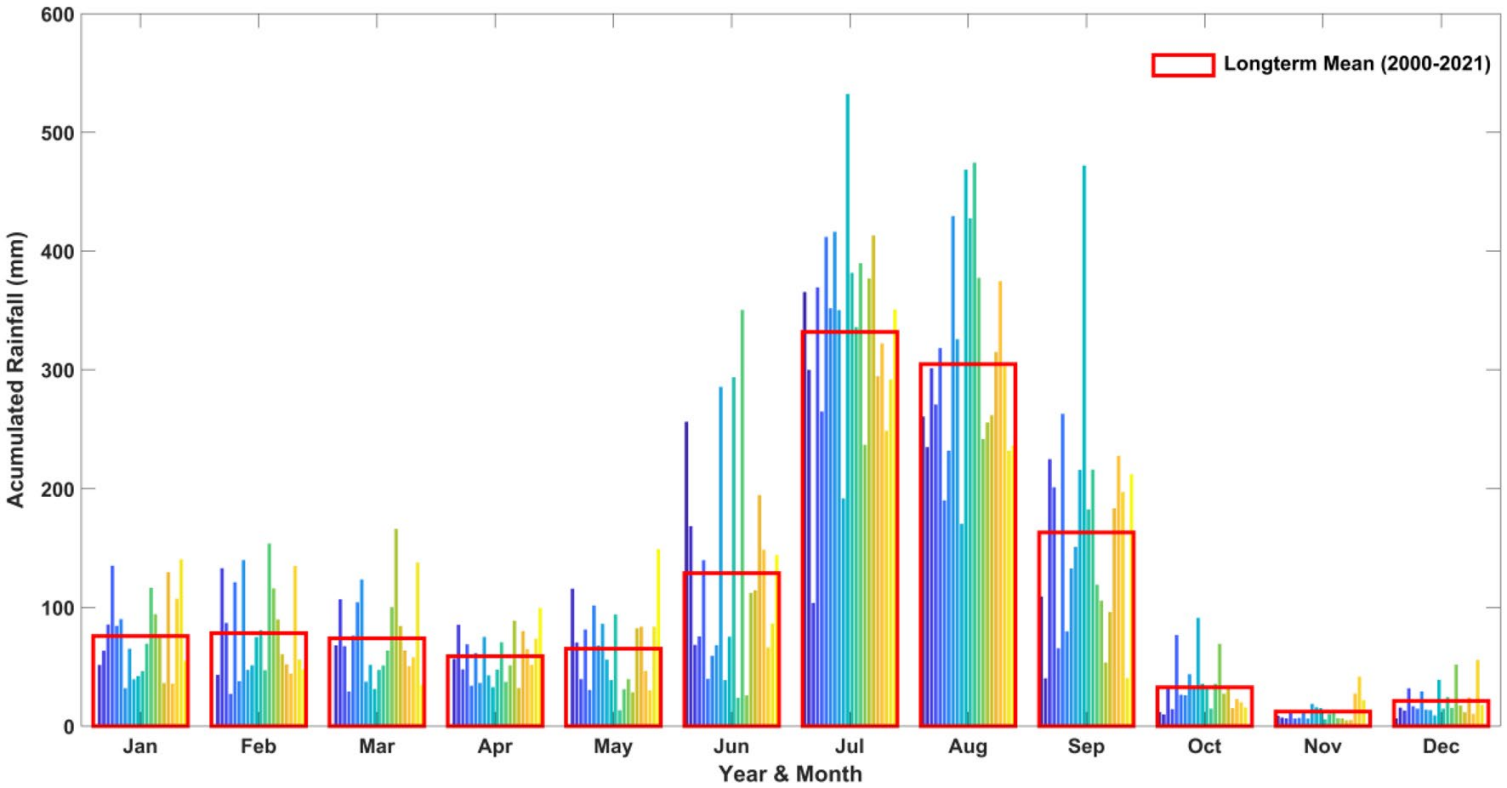
Accumulated rainfall (Long-term mean) over the Study area.

Figure 4 offers a visual representation of the long-term average of monthly recorded rainfall data in the study area from 2000 to 2021 to gain insight into the average rainfall during the same timeframe. The graph illustrates a significant rise in the average long-term rainfall within the study area. This increase is particularly notable during the months spanning from June to September. Notably, the figure underscores that during the years 2000 to 2021, the months of July and August in the area witnessed multiple heavy rainfall episodes due to monsoon. For these two months, the long-term average surpasses the 300 mm mark. In our results and discussion, we unravel the ramifications of persistent and substantial rainfall throughout these crucial months. The enduring deluge sets in motion a series of impactful consequences, ranging from escalated surface runoff and heightened river discharge to the looming specter of rapid flooding and landslides. This intricate web of effects intricately influences the stability of the soil, the vitality of vegetation, and the delicate balance within local ecosystems^71^. The findings highlighted in Fig. (3 and 4) underscore the critical significance of examining monthly rainfall data to comprehend the relationship with average monthly rainfall trends from (2000–2021) in the Himalayan region. The figure specifically draws attention to the months characterized by substantial rainfall, which may have result in disasters such as flash flooding and landslides. So we have concluded the study area may have received flash flooding by heavy rainfall during June to September (2000–2021).The daily rainfall data from 2001 to 2021 was allowed for non parametric trend analyses using Mann–Kendall test, Sen’s slope analysis. Modified Mann–Kendall and autocorrelation function for trend analysis.

Our analysis delved into daily rainfall data, downloaded from (ww.nasa.giovanni.com). We aimed to discern trends in key parameters, including monthly rainfall during the monsoon season (June to September), monsoon season data, annual rainfall, heavy rainfall events (> 50 mm/Day), and the number of wet days (> 2.5 mm/Day). Table 1 provides a comprehensive analysis of rainfall trends and extreme rainfall events from 2000 to 2021. In June, a negative autocorrelation was observed, and the findings are statistically significant at a 95% confidence level, so we considered modified MK test instead of original MK test. Employing the non-parametric Mann–Kendall test (MK/mMK) for trend analysis, our findings revealed a general non-significant increasing trend, with the exception of July, which exhibited a non-significant decreasing trend. Noteworthy was the significant increase in the number of wet days at a 0.05% significance level. Sen’s slope analysis further emphasized an annual increase in rainfall at a rate of 4.558 mm. These results provide valuable insights into the evolving rainfall patterns in the studied region, with implications for understanding climate variations.

**Table 1:** Trend analysis of rainfall and extreme rainfall 2000–2021.

Months	ACF	P	Z	Q
June	− 0.471	0.021	0.997	1.898
July	− 0.180	0.377	− 0.151	− 1.036
August	0.217	0.286	0.030	0.088
September	0.020	0.920	0.030	− 1.298
Monsoon	0.061	0.764	0.513	3.067
Annual	− 0.102	0.615	0.815	4.558
Number of heavy rainfall events > 50 mm/Day	− 0.341	0.094	0.831	0.000
Number of wet days (> = 2.5)/Day	0.013	0.951	2.206*	0.714

*Significant at 99% Confidence Interval.

### Topographic influence on rainfall and temperature over the study area

**Figure 5:**
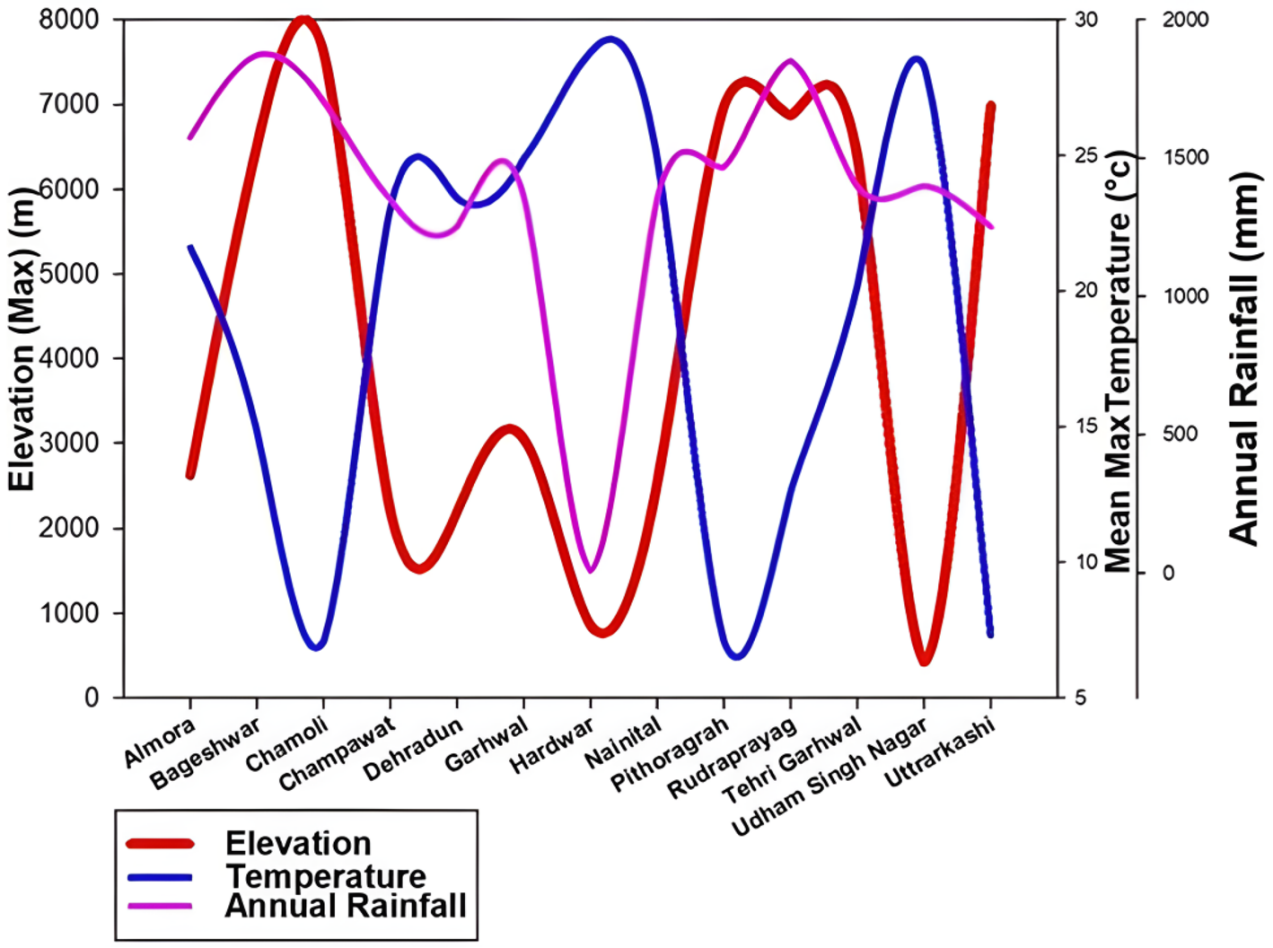
Topographic influence on the atmospheric parameter (Temperature and rainfall).

Exploring the realm of abundant rainfall at lofty Himalayan elevations delves into the captivating interplay between topography and the dynamic shifts in atmospheric parameters. Our investigation ventures beyond the surface, intricately analyzing the elevations across diverse districts within our study area. Figure 5 serves as a visual gateway, unraveling the fascinating discourse on how these elevational nuances weave a compelling narrative of change, orchestrating the dance between rainfall patterns and temperature shifts across our meticulously examined landscape. Using Fig. 5, we can correlate the significant relationship between the amount of rainfall and the topography over the Himalayan region of Uttarakhand. The figure distinctly delineates various districts of Uttarakhand, such as Bageshwar, Chamoli, Nainital, Pithoragarh, Rudraprayag, and Tehri Garhwal, positioned at elevations surpassing 7000 m. The presented data establishes a conspicuous correlation between the received rainfall and the elevated nature of these districts, showcasing those areas above 7000 m experience substantial annual rainfall exceeding 1500 mm. This correlation underscores the notable influence of elevation on the precipitation patterns in the Himalayan region. Higher elevations tend to attract more moisture from the atmosphere, leading to increased rainfall^72^**.**

Figure 5, in conjunction with the citation of Rafiq et al. 2016^73^, emphasizes the significant connection between mean maximum temperature and elevation within the Himalayan region. The figure illustrates that as elevation increases, there is a corresponding decline in mean maximum temperature. This well-known phenomenon is called the "lapse rate," which describes the temperature decrease with rising altitude. Areas above 7000 m experience notably lower temperatures than those at lower elevations. The lapse rate is a fundamental climatic characteristic particularly relevant in mountainous terrains like the Himalaya. As air ascends along the slopes, it cools down due to decreasing atmospheric pressure, forming clouds through condensation. These clouds subsequently contribute to rainfall, as discussed in the study by Wang Keyi et al.^72^. Higher elevations experience a more pronounced temperature decrease, resulting in elevated rainfall levels.

**Figure 6:**
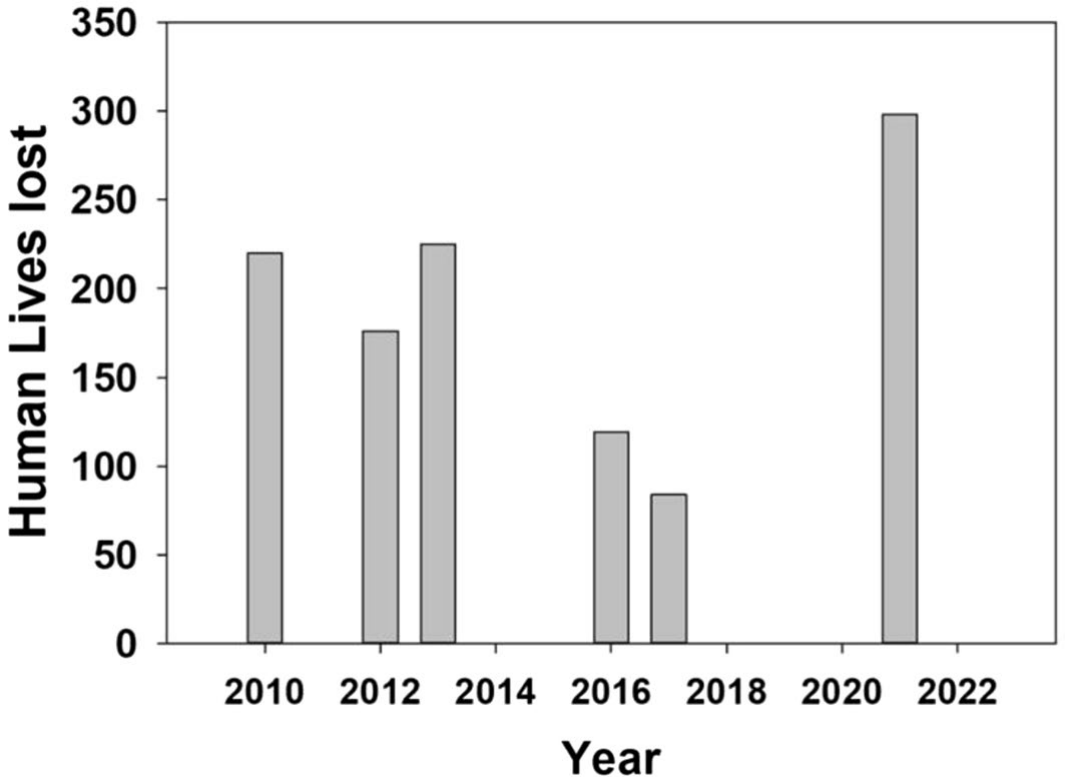
Number of human lives lost during heavy rainfall episodes in Uttrakhand.

The steep slopes in the Himalayan region significantly correlate with the number of casualties resulting from cloud bursts, landslides, and flash floods caused by heavy rainfall events. The presence of steep gradients exacerbates the impact of sudden and intense rainfall, leading to flash floods and landslides. Topography is crucial in disasters, particularly flash flooding and landslides, commonly observed in the Himalayan region^2^. These natural disasters have resulted in substantial loss of life and livelihood, as depicted in Fig. 6**.** Over 300 casualties were reported due to landslides, flash flooding, and cloud bursts in Uttarakhand during 2021. From 2010 and 2013, the loss was restricted to nearly 230 causalities each year. The Himalayan steep gradients are especially vulnerable to the effects of rainfall and climate change^74^.

Moreover, these mountainous regions' ecological and socioeconomic systems are becoming increasingly vulnerable due to the rising human population^2^. These disasters cause severe damage to infrastructure, properties, human lives, and the environment. Furthermore, they can exacerbate other hardships, including the spread of diseases, financial instability, environmental degradation, and social conflicts^74^.

In summary, the steep slopes in the Himalayan region play a critical role in the occurrence and severity of disasters such as flash floods and landslides. The susceptibility of these areas to heavy rainfall and climate impacts poses significant challenges for ecological and socioeconomic systems, particularly with the increasing human population. The aftermath of these disasters is far-reaching and extends beyond the immediate loss of life and property, affecting various aspects of human life and the environment in the region.

### Flash flood event during October 2021

**Figure 7:**
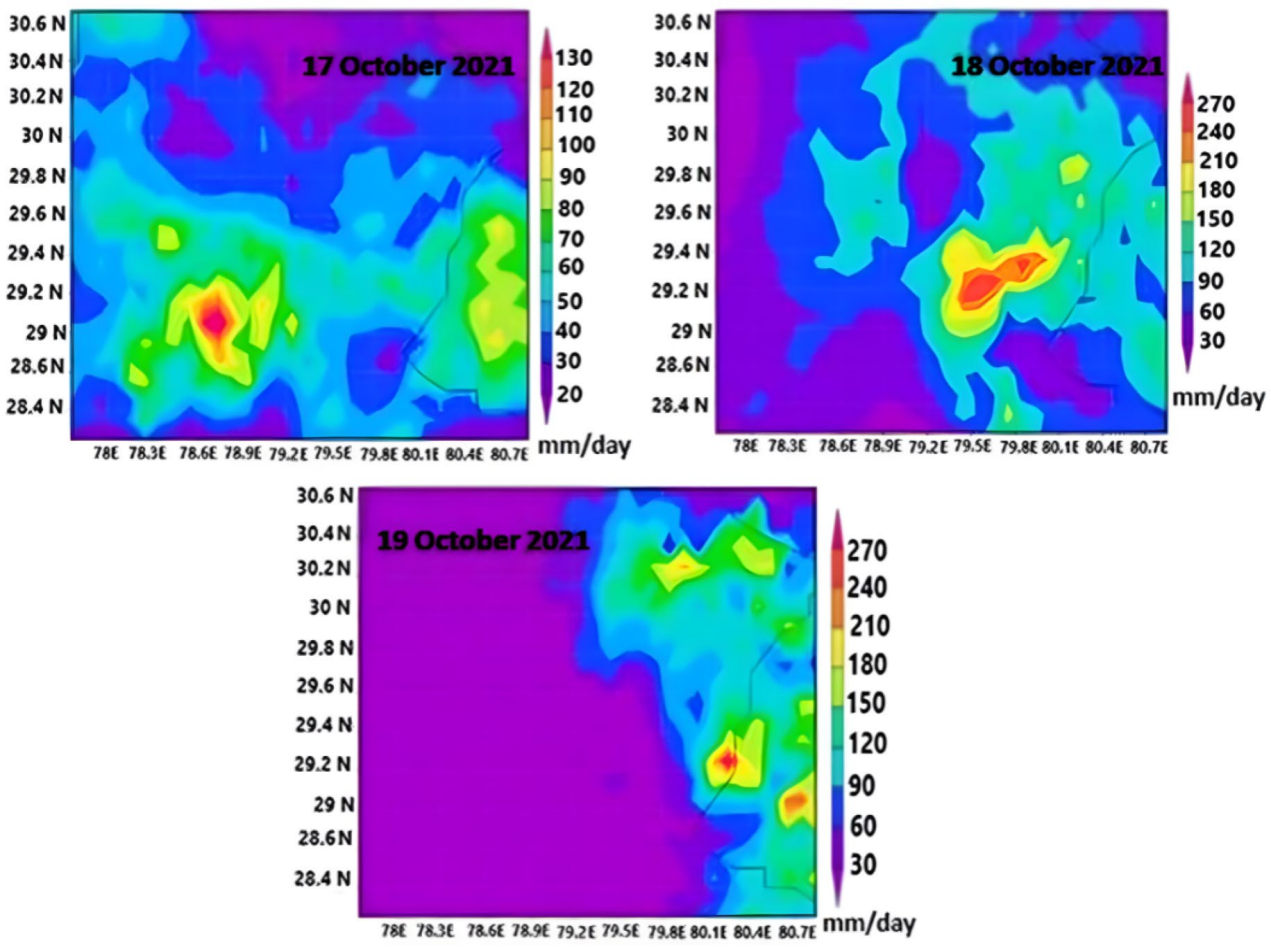
Time series heavy rainfall episodes over the Study area.

As delineated in Fig. 7, our investigation reveals a distinctive pattern in precipitation dynamics. Traditionally, the region encounters heightened rainfall exclusively from June to September, aligning with the monsoon season. Flash flooding, consequently, primarily manifests during this period. However, the anomalous occurrence in October 2021 is unprecedented in our dataset. For the first time, our analysis, depicted in Fig. 7, captures the manifestation of intense rainfall episodes leading to flash flooding in the Nainital region, Uttarakhand. As this was the rare case the study area has received heavy raifnall during month of october 2021. This may be due to western distribuance that area very rarely is receiving. The infrequency of such events in the area may be attributed to the rarity of western disturbances impacting the region. Utilizing the technique developed by Mishra^48^, we conducted the study to map daily monthly and spatial distribution of rainfall amount using Meteosat-8 data. The study employs real-time monitoring to track and analyze flash flooding, shedding light on the atmospheric parameters that contributed to the occurrence of this unique episode.

During October 2021, the region of Nainital, Uttarakhand experienced a series of rainfall events. From 12 to 15 September 2021, the area witnessed the development of low-pressure systems from the Bay of Bengal, as documented in the IMD Report 2021. This convergence of low-pressure systems led to several episodes of heavy rainfall over the Himalayan region^74^. Unfortunately, the consequences of these multiple rainfall episodes were severe, causing flash flooding and triggering landslides in various parts of the Indian Himalaya. Over the past few decades, there has been a noticeable upward trend in flash flooding incidents, particularly in the Himalayan region, which can be attributed to the effects of climate change^75^. As global temperatures rise and weather patterns become more erratic, the delicate balance of the Himalayan ecosystem is being disrupted, leading to intensified rainfall events and a higher risk of natural disasters like flash floods and landslides. These alarming changes underscore the urgent need for climate action and measures to address the impacts of climate change on vulnerable regions like the Himalaya. In October 2021, Nainital, Uttarakhand experienced an unusual and devastating flood event, an occurrence that is typically rare during this particular month. The torrential floodwaters swept away numerous homes and disrupted transportation networks, leaving the region in turmoil. In response to this calamity, various defence groups, such as the army and national defense forces, were promptly deployed to the Himalayan state to conduct rescue operations for residents and tourists. The impact of the flood was further exacerbated by landslides, which severed many districts from the rest of the region, as roads were blocked by mud and debris. The region's vulnerability to such natural disasters can be traced back to historical records, as it has been experiencing substantial rainfall since as early as 1857^76^. During 17th, 18th, and 19th of October 2021 a series of heavy rainfall episodes in Nainital, Uttarakhand, leading to flash flooding and landslides. The dire consequences resulted in widespread destruction of both lives and livelihoods^2^. Figure 7 highlights the visual representation of rainfall distribution over three days. The illustration provides valuable insights into the amount and pattern of rainfall that occurred during this critical period. Notably, the data reveals a remarkable occurrence on the 18th and 19th of October, where the study area experienced an abrupt 270 mm of rainfall. This substantial rainfall in just two days is an alarming and unprecedented event, signifying the intensity and severity of the weather system that hit the region. Moreover, it is essential to note that the 270 mm rainfall figure is not solely confined to those two days but is the cumulative result of heavy rainfall from multiple rainy spells that persisted during the specified period. The confluence of these rain events led to an overwhelming deluge, which became a primary driver of the extreme flooding that engulfed Nainital, Uttarakhand.

**Figure 8:**
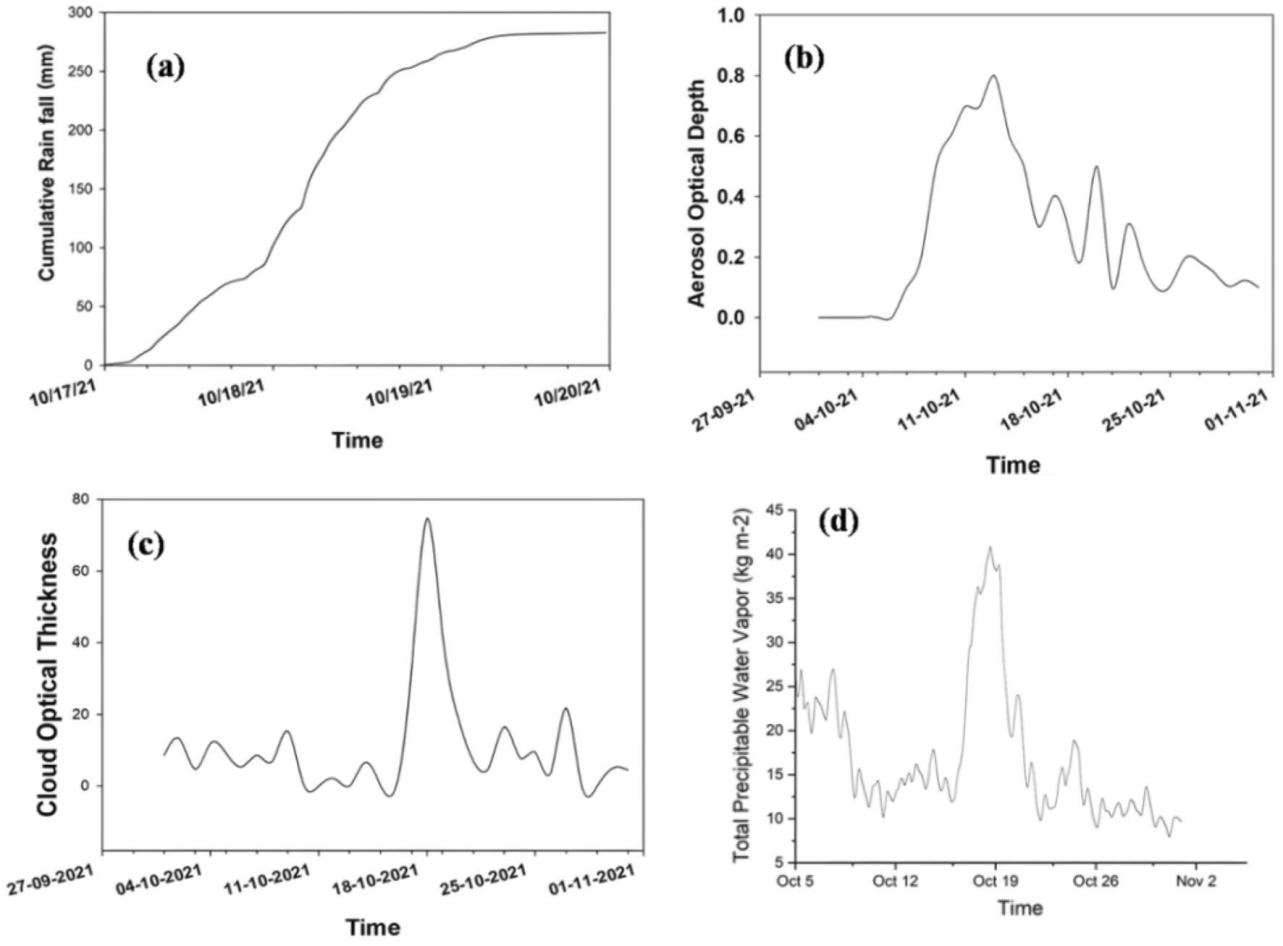
(**a**) Cumulative rainfall over the Nanital Utrankhand, (**b**) Aerosol optical depth over the Nanital Utrankhand, (**c**) Cloud optical thickness over the Nanital Utrankhand, (**d**) Total Perceptible water Vapor over the Nanital Utrankhand.

The analysis of near real-time monitoring of flash flooding in the area involved examining pre-flood atmospheric data related to aerosol optical depth, cloud optical thickness and total perceptible water vapour over the study area, as depicted in Fig. 8b,c,d. The study revealed a significant correlation between the pre-flood atmospheric data and the occurrence of extreme and multiple rainfall episodes in the region. This indicates that cloud formation and the presence of moisture are closely linked to the presence of aerosol particles^77^. The analysis of aerosol data in the study area revealed a significant presence of aerosol content in the atmosphere before the flood. This observation was particularly evident from the data recorded between the 5th and 8th of October 2021, as depicted in Fig. 8. The aerosol optical depth during this period was measured to be around 0.8, a noteworthy value for its potential impact in inducing heavy rainfall and flash flooding^78,79^. Aerosols are tiny particles suspended in the air, which can have important implications for weather and climate patterns^80^**.** High aerosol optical depth, as indicated by the measurement of 0.8, suggests a relatively dense concentration of aerosol particles in the atmosphere during the specified timeframe. Such high aerosol levels can act as cloud condensation nuclei, providing necessary sites for water vapour to condense and form cloud droplets. This phenomenon is crucial for cloud formation and rainfall processes^81^**.** The significance of aerosols in cloud formation lies in their ability to serve as nuclei for the aggregation of water vapour, leading to the development of clouds. This thick cloud cover resulted in considerable precipitable water vapour from the 17th to 19th of October, as shown in Fig. 8^82,83^. These atmospheric parameters resulted in favorable conditions for extreme with multiple rainfall episodes over the study area from 17 to 19th October 2021,finally, the extreme rainfall episodes attributed to flash flooding over the Nainital, Uttarakhand.

**Figure 9:**
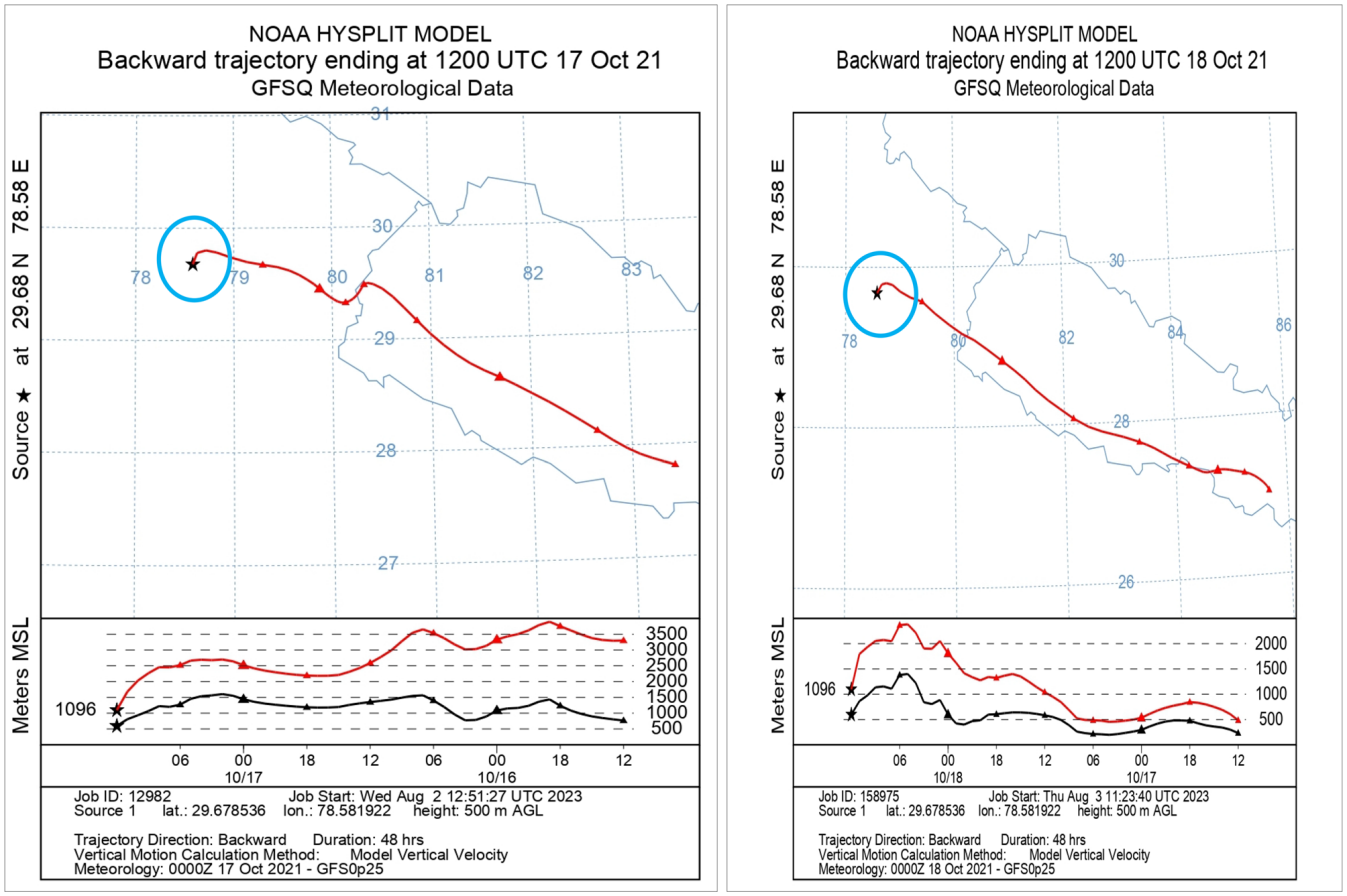
Backward trajectory of Aerosol during 17th, 18th and 19th October 2021 over the study area source encircled.

When moisture condenses around aerosol particles, it contributes to the formation of larger cloud droplets. These larger droplets can result in more intense rainfall events, potentially leading to flash flooding under certain conditions^82,83^. Furthermore, the HYSPLIT trajectory analysis revealed a profound influence of air masses originating or passing through western regions on the Himalayan radiation budget. This suggests that atmospheric dynamics from these areas significantly impact the weather patterns and climate in the Himalayan region. To gain deeper insights into the role of aerosols in the Himalayan radiation budget, the study also examined the Atmospheric Radiative Forcing (ARF)^14^. In the investigation of aerosol data, a backward trajectory analysis was conducted depicted in Fig. 9, focusing on the 17th and 18th of October 2021. The analysis aimed to trace the movement and direction of aerosols in the atmosphere 48 h before reaching the target area encircled in Fig. 9. The findings of figure demonstrated journey of aerosol during these days, shedding light on their movement and behavior in the study area. Specifically, on the 17th of October, the source of aerosols was observed at an altitude of 3500 m above Mean Sea Level (MSL). The tracked trajectory of aerosols reveals a gradual descent from an initial altitude of 3500 m above Mean Sea Level (MSL), ultimately reaching the research target at 1096 m MSL. This horizontal movement of aerosols suggests a potential influential role in the occurrence of heavy rainfall that result flash flooding over the study area by providing the favorable atmospheric conditions.

The comprehensive analysis conducted in this study has significantly advanced our understanding of the intricate interactions between various atmospheric parameters, aerosols, and rainfall patterns, all of which collectively contribute to heavy with multiple rainfall episodes that resulted flash flooding event in the Nainital region of Uttarakhand. The severity of such flash floods is starkly evident from the tragic loss of fifty lives and the extensive damage to property and infrastructure.

A key highlight of this study is the application of remote sensing data, including total aerosol optical depth, cloud cover thickness, total precipitable water vapour, and rainfall product (Meteosat-8), for real-time monitoring of flash floods. The use of cutting-edge satellite technology and geospatial data has proven to be pivotal in closely monitoring and tracking flash floods, enabling timely and efficient responses to mitigate the impact of these disasters. The findings of this research underscore the vital importance of leveraging advanced technology and scientific research to address the challenges posed by flash flooding in the Himalayan region. To effectively combat these challenges, a comprehensive and multi-faceted approach is imperative. This may encompass implementing measures to counteract the impact of climate change on weather patterns, advocating for sustainable land use practices to reduce vulnerability, and bolstering the resilience of critical infrastructure to withstand the impacts of extreme weather events like flash floods.

Furthermore, the study presents a unique occurrence in the Nainital region of Uttarakhand, Himalaya, wherein heavy rainfall, marked by multiple episodes, led to flash flooding during October 2021, an unusual event when compared to the time series precipitation analyzed in the study. The investigation emphasizes the significant role of elevation in influencing rainfall and temperature variations in the region. The study emphasizes the significance of continuous scientific research and monitoring efforts to gain invaluable insights into the underlying patterns and drivers of flash flooding in the Himalaya. Armed with this knowledge, authorities can formulate robust strategies and policies to minimize the impact of future flash floods and safeguard the lives and livelihoods of the communities residing in the region. This study reaffirms the crucial role that satellite data and geospatial technology play in effective disaster management. It underscores the urgency of adopting proactive measures to address the mounting risks of flash floods in vulnerable regions like Nainital, Uttarakhand. By synergizing scientific research, advanced monitoring techniques, and community engagement, authorities can work towards building a more resilient future, better equipped to respond to and mitigate the repercussions of flash flooding events.

## Conclusion

With their immense size and unique geological features, the Himalaya are prone to flash flooding incidents that pose significant risks to human life and infrastructure. Natural factors, such as tectonic activities and glacial lakes, and human-induced changes, including deforestation and land use alterations, influence these flash floods. In the Nainital region of Uttarakhand, the primary cause of flash floods is often attributed to cloud bursts accompanied by heavy rainfall episodes. The study highlights the crucial role of rainfall product and remote sensing data including total aerosol optical depth, cloud cover thickness and total precipitable water vapour, in real-time short-lived flash flood monitoring. The study emphasizes the significant role of elevation in influencing rainfall and temperature variations in the region. The application of satellite technology and geospatial data has proven to be instrumental in promptly tracking and responding to flash flood events. A comprehensive approach is necessary to address the challenges of flash flooding in the Himalaya. This may involve implementing measures to mitigate the impact of climate change, promoting sustainable land use practices, and enhancing infrastructure resilience. The study highlights a significant shift in precipitation patterns of Nainital, with usual heightened rainfall and flash floods. The rarity of such events in the region may be linked to infrequent western disturbances.

The research contributes valuable historical data and insights into the patterns of heavy rainfall and flash floods in the region. It underscores the alteration in precipitation patterns attributed to variations in atmospheric parameters over the study area. The findings demonstrate continuous monitoring and scientific research are critical for developing effective strategies to mitigate the impact of flash floods and safeguard communities in vulnerable regions like Nainital Uttarakhand. Overall, this study emphasizes the urgent need for climate action and proactive measures to address the rising risks of flash floods. By integrating advanced technology, scientific research, and community engagement, authorities can work towards building a more resilient future and better preparedness to tackle extreme weather events (Supplementary Information).

### Supplementary Information

Supplementary Information.

## Data Availability

The data that support the findings of this study are available from the corresponding author upon reasonable request.
